# Forgiveness of Sin

**Published:** 1889-10-12

**Authors:** 


### Words of Consolation.

### FORGIVENESS OF SIN.

When the careless sinner has been awakened from his indifference, and his hard and impenitent heart softened by sickness, he begins to get uneasy and turns on all sides for relief. He cannot see his way out of the discomfort and misery which a conviction of sin brings in its train. He cannot go forward, he dare not go back. Then he tries to pray; and, alas! his prayer too often is only a feeble moan of agony and dumb expostulation with the Almighty against his sufferings; or he thinks he is not worthy to be heard by God, and says, Who am I that my prayers should prevail with Him? But no prayers were ever accepted for their worthiness. If he has faith enough to say "Lord, I believe; help, Thou, my unbelief"; or, "God be merciful to me, a sinner"! He who sees all hearts, and knows their weakness, will send an answer of peace.

If we are overwhelmed by the weight of our sins, and cruelly torn by remorse, let us hasten, with Mary Magdalene, to find Jesus. She, poor sinner, heard that He was at the Pharisee's house. He who was so good, who healed the sick, who comforted the afflicted, who did good to the whole world! She went and cast herself at His feet, and there wept. What comfort thus to weep at the feet of Jesus, saying nothing, asking nothing, but contented to weep and make known by our tears our repentance and our love.

But Mary did not stop here. She longed to show her love by actions, so she washed Christ's feet with her tears and wiped them with the hairs of her head, and breaking a box of very precious ointment, lavished the rich contents till the whole house was filled with the fragrant perfume. Here was the entire rendering of self to God. She worshipped and served Him with her body, her soul, and her money. And what a blessed return our Lord vouchsafed. He who came to seek and to save that which was lost graciously accepted these marks of penitence and love.

He shielded Mary from the charge of extravagance, and the blame cast on her by the Pharisee, in these words: "Her sins, which were many, are forgiven her, for she loved much." Yes, it is love for God which makes everything easy both in heaven and earth. If we will only try to love God with all our hearts, with all our mind, with all our soul, and with all our strength, we, too, shall hear the gracious words, Thy sins are forgiven thee. And now, being freely forgiven, Mary kept close to Jesus; she followed Him wherever he went, and ministered to his necessities. She, with the other women who loved our Lord, was the last at the cross, the first at the tomb. They saw Him at night laid in the rock, and at early dawn hastened with sweet spices to embalm His body. With a dim, blind confusion of heart they struggled on, but when they arrived at the grave, O, comfort for sorrowing hearts, O, wonder and joy, they found an open sepulchre, an angelic vision, and the earliest tidings of the risen Lord.

